# Zoliflodacin for uncomplicated gonorrhea: clinical evidence and implications beyond ceftriaxone plus azithromycin

**DOI:** 10.1097/MS9.0000000000005136

**Published:** 2026-05-14

**Authors:** Aliza Ahmed, Mubashira Noor, Yumna Shahzad, Aleeza Abid, Muhammad Hassan Saeed, Ihsan Qamar

**Affiliations:** a Department of Medicine and Surgery, Jinnah Sindh Medical University, Karachi, Sindh, Pakistan; b Department of Medicine and Surgery, National Institute of Cardiovascular Diseases, Karachi, Sindh, Pakistan; c Department of Medicine and Surgery, Jinnah Postgraduate Medical Centre, Karachi, Sindh, Pakistan; d Department of Medicine and Surgery, Spinghar Medical University, Kabul, Afghanistan

**Keywords:** antimicrobial resistance, antimicrobial stewardship, azithromycin, ceftriaxone, DNA gyrase inhibitors, Neisseria gonorrhoea, oral antibiotic therapy, sexually transmitted infections, zoliflodacin

## Abstract

**Background:**

Globally, gonorrhea remains a common sexually transmitted infection that can lead to serious sexual and reproductive health complications if left untreated. Management has become more challenging due to the rapid progression of antimicrobial resistance in *Neisseria gonorrhoeae*. Currently, ceftriaxone-based regimens continue to be the standard treatment; however, the sudden demand for new treatment options is highlighted by concerns regarding resistance, the requirement for intramuscular administration, and the declining effectiveness of azithromycin.

**Objective:**

This review aims to evaluate the emerging role of zoliflodacin in the treatment of gonorrhea, with an emphasis on its potential to serve as a resistance-focused alternative, its oral administration, and its novel antimicrobial target.

**Methods:**

A narrative literature search was conducted using PubMed, Google Scholar, and Scopus to identify relevant studies published between 2016 and 2026. Randomized clinical trials, systematic reviews, narrative reviews, and international guidelines from the WHO, ECDC, and CDC were included. Literature selection was guided by SANRA.

**Results:**

A single 3-g oral dose of zoliflodacin appears to exhibit high microbiological cure rates for uncomplicated urogenital and rectal gonorrhea, and it is not less effective than ceftriaxone plus azithromycin for urogenital infections, according to Phase 2 and recent global Phase 3 clinical trials, which reported mild gastrointestinal side effects. Its mechanism of action provides limited evidence of resistance and is effective against multidrug-resistant *Neisseria gonorrhoeae*.

**Conclusion:**

Zoliflodacin represents a potentially effective oral alternative to the current ceftriaxone-based regimen for gonorrhea. With routine observation and careful stewardship, these findings may contribute significantly to the expansion of future treatment strategies.

## Introduction

*Neisseria gonorrhoeae* is a prevalent sexually transmitted bacterium, with an estimated 87 million new adult cases in 2016, representing a 12% increase from 2012[1]. Gonorrhea is associated with substantial morbidity, including pelvic inflammatory disease, infertility, ectopic pregnancy, and adverse pregnancy outcomes, and it contributes to increased HIV transmission[2].

HIGHLIGHTS

- Zoliflodacin is an emerging oral therapy that offers a practical alternative to injectable ceftriaxone for uncomplicated gonorrhea. In Phase 3 trials, a single 3-g oral dose showed non-inferior urogenital cure rates when compared to ceftriaxone plus azithromycin.
- Its distinct GyrB inhibition mechanism continues to be effective against *Neisseria gonorrhoeae*, which is resistant to multiple drugs. Zoliflodacin shows good tolerability, with mainly mild gastrointestinal adverse effects and no injection-site complications.
- This therapy reduces reliance on cephalosporins and macrolides amid rising resistance and supports antimicrobial stewardship. Ongoing surveillance, real-world studies, and resistance monitoring are needed to guide policies and ensure the safe future use of zoliflodacin.

The World Health Organization (WHO) and US Centers for Disease Control and Prevention (CDC) now classify *N. gonorrhoeae* as a high-priority or urgent threat pathogen because of its escalating antimicrobial resistance (AMR), which has rendered sulfonamides, penicillins, tetracyclines, fluoroquinolones, and many macrolides ineffective and reduced susceptibility to extended-spectrum cephalosporins^[^2,3^]^. Although ceftriaxone-based regimens currently retain high efficacy in many regions, ceftriaxone-resistant and extensively drug-resistant strains, including the internationally disseminated FC428 clone, have been documented across several continents^[^1,4^]^. The 2021 CDC recommendations favor 500 mg intramuscular ceftriaxone monotherapy, plus doxycycline when chlamydial infection has not been excluded, while acknowledging the absence of reliable alternatives for pharyngeal disease and for patients with severe cephalosporin allergy[5].

Ceftriaxone-based therapy has several limitations. It requires parenteral administration by trained staff, complicating delivery in low-resource or community settings^[^2,6^]^. Additionally, the global rise in azithromycin minimum inhibitory concentrations and treatment failures has eroded the rationale for dual ceftriaxone–azithromycin therapy and raised concerns about collateral selection for macrolide resistance in both gonococci and commensal *Neisseria* species^[^3,5^]^. Together, these factors highlight an urgent need for novel, preferably oral, agents with no cross-resistance to existing classes, robust activity at all anatomic sites, and properties compatible with antimicrobial-stewardship goals^[^2,4,6^]^.

Zoliflodacin, a first-in-class oral spiropyrimidinetrione, selectively inhibits DNA gyrase subunit B via a distinct binding mode different from fluoroquinolones and shows strong activity against drug-resistant *N. gonorrhoeae*, with low resistance rates and good pharmacokinetics for single-dose administration^[^1,4^]^. Phase 2 and recently completed global Phase 3 trials have demonstrated the non-inferiority of single-dose zoliflodacin to standard ceftriaxone plus azithromycin for uncomplicated gonorrhea, positioning it as a promising future alternative to ceftriaxone-based regimens^[^7,8^]^.

In the context of growing gonococcal AMR, this review aims to compare zoliflodacin with ceftriaxone plus azithromycin, evaluating its efficacy, safety, and tolerability, and exploring its implications for antimicrobial stewardship and future global treatment strategies.

## Methods/literature search

A structured search was conducted across PubMed, Google Scholar, and Scopus to identify relevant publications from 2016 to 2026. The Boolean combinations of keywords in the search strategy included “zoliflodacin,” “gonorrhoea,” “*Neisseria gonorrhoeae*,” “Phase 2 trial,” “Phase 3 trial,” “antimicrobial resistance,” and “ceftriaxone.”

Eligible sources included peer-reviewed clinical trials, systematic reviews, key narrative overviews, and guideline or policy documents from WHO, CDC, and ECDC addressing zoliflodacin’s role in gonorrhea treatment, prioritizing randomized controlled trials reporting efficacy, safety, and resistance outcomes.

Non-human studies, early *in vitro* work without clear clinical translation, and reports lacking primary clinical data were excluded. This paper adopts a narrative approach rather than a systematic approach, and the selection of literature was guided by the Scale for the Assessment of Narrative Review Articles (SANRA) 2.0 to ensure balance and accuracy.

The final selection emphasizes studies that explore the mechanisms of action, clinical efficacy, tolerability, resistance and stewardship implications, as well as public health and implementation relevance.

## Current standard of care: ceftriaxone plus azithromycin

Azithromycin (AZM), also known as a second-generation macrolide, is readily taken up by various cell types, including fibroblasts and leukocytes. The key mechanism of action of AZM involves the inhibition of bacterial protein synthesis by binding to the 50S ribosomal subunit, partly obstructing the nascent peptide exit tunnel. AZM is effective *in vitro* against various pyogenic pathogens, e.g., *N. gonorrhoeae* and *Moraxella catarrhalis*, as well as β-lactam–resistant organisms, including *Legionella* and *Chlamydia* species. Azithromycin resistance in *N. gonorrhoeae* originates from mutations that lead to the overexpression of the MtrCDE efflux pump and alterations in the 23S rRNA target, decreasing the drug's binding ability. The reason behind these mechanisms involves suboptimal dosing and extended or inappropriate azithromycin use[9].

Ceftriaxone, a third-generation cephalosporin, acts by inhibiting bacterial cell wall formation by binding to penicillin-binding proteins, resulting in bactericidal activity and having a long half-life compared to other cephalosporins. This property demonstrates high potency against *N. gonorrhoea* and features favorable pharmacokinetics[10].

The historical rationale for using azithromycin and ceftriaxone emerged due to widespread resistance against earlier antibiotics, e.g., penicillins, tetracyclines, and fluoroquinolones. Combining two agents with distinct mechanisms of action aimed to improve microbial clearance and limit the emergence of resistance[11].

However, emerging resistance trends are increasingly undermining this approach. Recent studies show a substantial rise in AZM resistance reported in multiple regions, including Europe and the Western Pacific, characterized by increasing inhibitory concentrations and verified treatment failures, raising concerns about its continued role in gonorrhea management[3]. On the other hand, threats to ceftriaxone longevity have become evident. Emerging studies highlight decreased susceptibility and ceftriaxone-resistant strains, indicating the vulnerability of relying on a single remaining highly effective agent. However, ceftriaxone therapy is still rare but alarming[12].

Azithromycin is considered safe and well-tolerated but can cause gastrointestinal dysfunctions, rare hepatotoxicity, hypersensitivity, and potential QTc prolongation with arrhythmia risks, mainly in high-risk patients. Ceftriaxone is usually safe and well-tolerated, although it might cause gastrointestinal symptoms, hypersensitivity, and reversible biliary pseudolithiasis; some rare conditions include hemolytic anemia and hepatobiliary diseases[13]. The most common side effects of ceftriaxone therapy are diarrhea, exanthema, rash or pruritus, and reactions at the injection site, such as phlebitis and pain on intramuscular injection.

To cope with these emerging challenges, major international guidelines, such as the WHO and CDC, prioritize ceftriaxone monotherapy as the initial therapy for uncomplicated *N. gonorrhoeae* infections, though dual therapy with azithromycin is no longer routinely administered due to rising concerns about azithromycin resistance. However, in some cases, the WHO still allows dual therapy in limited settings lacking surveillance data^[^14,15^]^.

These findings highlight the urgent need for the development of new treatments, paving the way for agents such as zoliflodacin in the clinical management of uncomplicated gonorrhea.

## Zoliflodacin: mechanism and pharmacologic rationale

Zoliflodacin, also referred to as AZD0914 or ETX0914, is an investigational spiropyrimidinetrione antimicrobial agent that has been designated by the Food and Drug Administration (FDA) as a “qualified infectious disease product” and subsequently as a “fast track” agent for development exclusively as an oral treatment for gonococcal infections. In contrast to other treatments now on the market, zoliflodacin works by stopping the cleaved covalent gyrase complex and the creation of fused circular DNA, which are necessary for microbial production. The susceptibility of fluoroquinolone-resistant and vancomycin-resistant *Staphylococcus aureus* isolates to zoliflodacin, as well as ciprofloxacin-resistant and ceftriaxone-resistant *N. gonorrhoeae*, has demonstrated the efficacy of this mechanism. *Chlamydia trachomatis, Chlamydophila pneumoniae, Mycoplasma genitalium*, and *Ureaplasma* species are also susceptible to zoliflodacin[16].

Members of this medication class do not exhibit balanced targeting between gyrase and topoisomerase IV, which has exacerbated target-mediated fluoroquinolone resistance.

A single gyrase mutation is frequently sufficient to either (1) induce enough fluoroquinolone resistance to allow bacterial cells to escape drug toxicity or (2) allow bacteria to acquire additional mutations in either gyrase or topoisomerase IV and evolve into highly resistant infections. This unbalanced targeting has a profound effect on the development of drug resistance in two ways[17].

The majority of adverse events that were reported were gastrointestinal. Zoliflodacin was less successful in treating pharyngeal infections, but it showed promise in treating urogenital and rectal gonococcal infections. This restriction aligns with earlier guidelines for other medications, including spectinomycin and other fluoroquinolones[2].

By analyzing the pharmacodynamics of zoliflodacin in combination therapy with doxycycline against *N. gonorrhoeae* in our dynamic *in vitro* gonococcal HFIM, we were able to show that combination therapy involving zoliflodacin (0.5–4 g single dose) and doxycycline (200 mg, divided into 100 mg twice a day for 7 days) exhibits high efficacy when compared to zoliflodacin monotherapy[4].

## Clinical evidence for zoliflodacin

### Phase 2 randomized trial

The pivotal Phase 2 trial was a multicenter, randomized, open-label study comparing single-dose oral zoliflodacin (2 or 3 g) with 500 mg intramuscular ceftriaxone for uncomplicated urogenital gonorrhea[17]. In total, 179 participants (167 men, 12 women) were enrolled across U.S. sites from 2014 to 2015[17]. The microbiological intention-to-treat (micro-ITT) population included 141 participants with culture-confirmed urogenital *N. gonorrhoeae*^[^2,17^]^ with specimens collected from urethral/cervical, rectal, and pharyngeal sites at enrollment and a test-of-cure visit 6 ± 2 days post-treatment[17].

Zoliflodacin was formulated as a frozen oral suspension and administered under direct observation in 2- and 3-g single doses[17]. The 3-g dose was selected based on preclinical pharmacokinetic/pharmacodynamic work and hollow-fiber infection models, which suggested that ≥3 g effectively eradicates strains with pre-existing GyrB mutations and suppresses resistance emergence^[^4,7^]^. The trial used a 70:70:40 randomization (2 g: 3 g: ceftriaxone) for dose–response optimization while maintaining a robust comparator arm[17].

The primary endpoint of urogenital microbiological cure at test-of-cure in the micro-ITT population showed 96% cure in participants receiving 2- and 3-g doses of zoliflodacin, and 100% cure with ceftriaxone^[^2,17^]^. All rectal infections were cured with both zoliflodacin doses and ceftriaxone^[^2,17^]^. Pharyngeal infections showed variable outcomes: 50% cure with 2 g, 82% with 3 g, and 100% with ceftriaxone^[^2,17^]^, indicating high efficacy for urogenital and rectal disease but reduced reliability for pharyngeal infections, consistent with other emerging agents^[^2,18^]^.

Overall, 84 adverse events were reported: 24 in the 2-g group, 37 in the 3-g group, and 23 with ceftriaxone[17]; 21 events related to zoliflodacin were predominantly mild–moderate gastrointestinal symptoms^[^2,17^]^. No serious drug-related events or clinically significant safety signals were identified, and Phase 2 data supported further development and higher 3 g dosing for Phase 3^[^4,17^]^.

### Phase 3 randomized controlled trial

Zoliflodacin’s global, randomized, active-controlled, open-label Phase 3 non-inferiority trial (NCT03959527), sponsored by GARDP and partners, evaluated its efficacy against standard dual therapy for uncomplicated urogenital gonorrhea (with or without extragenital involvement), consistent with WHO-recommended first-line treatment^[^4,7,8^]^. Adults received either a single 3-g oral dose of zoliflodacin or standard dual therapy: ceftriaxone 500 mg intramuscularly plus azithromycin 1 g orally, with a non-inferiority margin of 12%, aligned with contemporary regulatory guidance for gonorrhea trials^[^4,7,19^]^.

The primary endpoint was microbiological cure at the urogenital site in the micro-ITT population at test-of-cure (days 4–8), defined as culture-confirmed eradication of *N. gonorrhoeae*^[^4,7,8^]^. Zoliflodacin achieved a 90.9% urogenital cure rate compared to 96.2% for ceftriaxone–azithromycin, with a non-inferiority margin of −5.3% (95% CI: 1.38–8.65 within the 12% and FDA 10% margins)^[^4,7,8^]^. Rectal and pharyngeal cure rates were reported as comparable between arms as secondary endpoints, indicating improved extragenital performance compared with Phase 2, likely reflecting the higher 3 g dose and optimized exposure^[^4,7^]^. A systematic review of rectal gonorrhea trials categorized zoliflodacin as an “emerging” agent with high, though slightly lower, pooled efficacy than current ceftriaxone-based regimens, underscoring the need for more extragenital data but supporting its clinical potential[18].

A zoliflodacin dose of 3 g was generally well tolerated in Phase 3 trials, with adverse event rates and profiles similar to those of ceftriaxone plus azithromycin, and dominated by mild gastrointestinal complaints. No deaths or treatment-related serious adverse events were reported^[^4,7^]^. Pharmacometric analyses indicated that a single 3-g dose achieves ≥96% probability of pharmacodynamic target attainment at MIC ≤0.25 µg/mL, covering virtually all isolates from surveillance and trial populations^[^20,21^]^. There was no clear on-treatment selection of high-level zoliflodacin resistance; global surveillance and WHO Enhanced Gonococcal Antimicrobial Surveillance Programme data continue to show high susceptibility in GyrB, with rare D429N variants and modest MIC elevation^[^7,22^]^. Dynamic hollow-fiber studies confirm that adequate 3 g exposure, with or without doxycycline, can eradicate predisposed strains and suppress resistance emergence^[^4,20^]^.

Overall, data from Phases 2 and 3 demonstrate that single-dose oral zoliflodacin achieves high urogenital and rectal cure rates, comparable to ceftriaxone plus azithromycin, with acceptable tolerability and a low resistance risk during treatment, positioning it as a leading oral alternative in the face of escalating cephalosporin-based limitations^[^2,4,7,8,17,20–22^]^.

**Figure 1.**
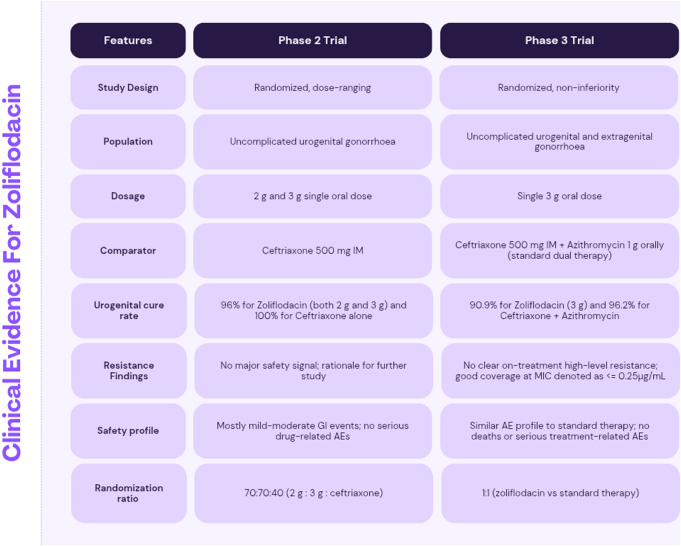
Comparison of Phase 2 and Phase 3 clinical trial parameters and outcomes for zoliflodacin in the treatment of gonorrhea. The table outlines the evolution of clinical evidence for zoliflodacin, transitioning from a dose-ranging Phase 2 study focused on urogenital infections to a large-scale, non-inferiority Phase 3 trial encompassing both urogenital and extragenital populations. Key highlights include the transition to a single *3 g oral dose*, comparable *urogenital cure rates* (90.9% in Phase 3) relative to standard dual therapy, and a consistent *safety profile* with no serious treatment-related adverse events (AEs) reported across both phases.

A comparative overview of Phase 2 and Phase 3 clinical trial parameters and outcomes is summarized in Figure 1.

## Comparative clinical interpretation

Zoliflodacin is considered an optimistic alternative to the current standard therapy for uncomplicated gonorrhea, which consists of administering intramuscular ceftriaxone paired with oral azithromycin[23]. In Phase 2 and 3 clinical trials, zoliflodacin showed comparable efficacy to the current first-line therapy, specifically for urogenital infections. The microbiological cure rate of zoliflodacin is similar to those achieved with ceftriaxone-based regimens, highlighting its promising role and making it an effective first-line or second-line agent. However, somewhat lower potency has been observed in pharyngeal infections, a limitation common to several existing antimicrobial agents. Overall, the current evidence indicates that zoliflodacin appears to be a clinically meaningful alternative due to the rising resistance to cephalosporins and macrolides^[^2,4,7,8,17,20–22^]^.

In the context of safety and tolerability, zoliflodacin has demonstrated favorable and tolerable outcomes similar to standard therapy. The reported adverse events included mild to moderate gastrointestinal symptoms, headache, neutropenia, and leukopenia. No serious adverse events have been observed. However, zoliflodacin has not been associated with any injection-site reactions, as compared to intramuscular ceftriaxone, which may improve patient comfort and acceptance^[^17,24^]^.

A key advantage of zoliflodacin is its oral route of administration, in contrast to ceftriaxone, which requires parenteral administration in a clinical setup. This simple treatment delivery reduces dependence on trained healthcare personnel and improves treatment availability in resource-limited areas. With easier treatment options, we can improve treatment adherence and outcomes, particularly for vulnerable populations^[^17,20^]^.

Zoliflodacin highlights important implications for antimicrobial stewardship. As a novel spiropyrimidinetrione targeting bacterial type II topoisomerases with binding sites in bacterial gyrase, which ultimately inhibits the formation of fused circular DNA and hence blocks its biosynthesis, it offers an opportunity to diversify therapeutic options and reduce selective pressure on existing drug classes^[^17,20,25^]^. In order to maximize its longevity, Zoliflodacin must be used cautiously and judiciously.

## Resistance, surveillance, and public health implications

As *N. gonorrhoeae* develops more resistance to antibiotics, the treatment and management of gonorrhea are at risk. All previously utilized classes of antibiotics that were advised for therapy have developed resistance. The last-line alternatives for empirical treatment, ceftriaxone and especially azithromycin, have seen an increase in *in vitro* and clinical resistance throughout the past 10 years[4].

Combined with the encouraging outcomes of the recent global zoliflodacin Phase 3 RCT for the treatment of uncomplicated gonorrhea, the high *in vitro* susceptibility among modern *N. gonorrhoeae* isolates (*n* = 2993) collected from eight WHO Enhanced Gonococcal Antimicrobial Surveillance Programme (EGASP) countries in the WHO Southeast Asian Region (Indonesia and Thailand), WHO Western Pacific Region (Cambodia, the Philippines, and Vietnam), and WHO African Region (Malawi, South Africa, and Uganda) during 2021–2024 supports additional clinical development, registration, and introduction of zoliflodacin for the treatment of uncomplicated gonorrhea[7].

## Limitations of current evidence

A Phase 2 randomized trial of zoliflodacin versus ceftriaxone included 179 participants, with few women or rectal cases[17]. The completed pivotal Phase 3 non-inferiority RCT lacks peer-reviewed data, relying on press releases, conference reports, or narrative overviews rather than published trial reports^[^2,8^]^.

Phase 2 and 3 summaries emphasize urogenital outcomes; low rectal and pharyngeal infection numbers limit efficacy precision[17]. Concerns persist regarding reduced pharyngeal cure and insufficient PK/PD and clinical data at extragenital sites^[^1,26,27^]^.

Surveillance indicates high susceptibility and conserved GyrB, but resistant or predisposed strains (e.g., GyrB S467N, D429N) can emerge under suboptimal exposure^[^1,26^]^, lacking long-horizon, post-introduction data on resistance evolution or transmission dynamics^[^1,2,26^]^.

Existing evidence comes from controlled trials and experimental PK/PD models^[^1,17^]^. No studies from routine STI services have yet evaluated real-world effectiveness, adherence, reinfection, or impact on antimicrobial stewardship and ceftriaxone-sparing strategies^[^2,8^]^.

## Future directions

Surveillance studies, both ongoing and planned, play an important role in monitoring zoliflodacin susceptibility and identifying resistance trends, as marked by recent WHO EGASP data demonstrating high susceptibility, although occasional increases in minimum inhibitory concentrations among isolates from Cambodia emphasize the need for continuous global resistance monitoring[7]. International initiatives led by the WHO and the CDC are expected to integrate zoliflodacin into existing gonococcal antimicrobial surveillance frameworks, enabling timely data collection and analysis.

Among the growing list of novel treatments for gonorrhea, zoliflodacin emerges alongside other potential treatments, such as gepotidacin, contributing to a growing range of options for combating multidrug-resistant *N. gonorrhoeae*[28]. To maximize their impact, it is essential to compare their mechanisms, effectiveness, and resistance patterns to guide appropriate sequencing or potential combination strategies, offering opportunities for diversified treatment approaches and reduced reliance on existing drug classes.

Importantly, zoliflodacin holds the potential to replace the current dual standard therapy. Its oral route of administration, single-dose treatment plan, and unique mechanism of action may allow for simplified monotherapy in selected cases, thereby reducing unnecessary antimicrobial exposure and resistance^[^7,24^]^. Persistent incorporation of surveillance findings with treatment strategy updates will be necessary for enhancing its clinical impact alongside safeguarding stewardship.

## Conclusions

Similar to fluoroquinolones, SPTs interact with the cleavage complex and affect gyrase/topoisomerase IV activity in two ways. Initially, they increase equilibrium concentrations of enzyme-generated DNA breaks by stabilizing the cleavage complex. The enzymes interact with two fluoroquinolone and SPT molecules, one of which inserts at each scissile link. As a result, these medications mostly promote double-strand breaks mediated by gyrase/topoisomerase IV. Second, fluoroquinolones and SPTs prevent gyrase and topoisomerase IV from completing their catalytic cycles by stabilizing cleavage complexes. Consequently, these medications also reduce the two enzymes’ total catalytic activity[17].

Target-mediated SPT resistance may potentially restrict the therapeutic effectiveness of this antibacterial family, even though zoliflodacin predominantly targets gyrase and seems to be less mutagenic than fluoroquinolones[17].

Recently, a global phase 3 randomized controlled clinical study (RCT) (ClinicalTrials.gov Identifier: NCT03959527) concluded the evaluation of zoliflodacin as a new treatment for uncomplicated gonorrhea. When compared to the internationally recommended treatment, which consists of ceftriaxone 500 mg single intramuscular dose plus azithromycin 1 g single oral dose, zoliflodacin 3 g single oral dose met the pre-specified statistical test for non-inferiority in this Phase 3 RCT (5.31%, 95% CI: 1.38–8.65%). The non-inferiority of zoliflodacin was shown to be within the predetermined 12% margin, as well as the 10% margin that the U.S. FDA had set[4].

Further assessments will be necessary before zoliflodacin is used to treat gonorrhea in the future. These assessments should look into potential antagonistic or synergistic interactions with other antibiotics that may be used in conjunction to treat gonorrhea, other concurrent STIs, or other infectious disorders[4].

## Data Availability

Not applicable.

## References

[1] Jacobsson S Golparian D Oxelbark J. Pharmacodynamic evaluation of dosing, bacterial kill, and resistance suppression for zoliflodacin against *Neisseria gonorrhoeae* in a dynamic hollow fiber infection model. Front Pharmacol 2021;12:682135. 34093206 10.3389/fphar.2021.682135 PMC8175963

[2] Raccagni A Ranzenigo M Bruzzesi E. *Neisseria gonorrhoeae* antimicrobial resistance: the future of antibiotic therapy. J Clin Med 2023;12:7767. 38137836 10.3390/jcm12247767 PMC10744250

[3] Derbie A Mekonnen D Woldeamanuel Y. Azithromycin resistant gonococci: a literature review. Antimicrob Resist Infect Control 2020;9:138. 32811545 10.1186/s13756-020-00805-7 PMC7436955

[4] Jacobsson S Golparian D Oxelbark J. Pharmacodynamics of zoliflodacin plus doxycycline combination therapy against *Neisseria gonorrhoeae* in a gonococcal hollow-fiber infection model. Front Pharmacol 2023;14:1172589. 10.3389/fphar.2023.1291885 PMC10733441 38130409

[5] Barbee LA St Cyr SB. Management of Neisseria gonorrhoeae in the United States: summary of evidence from the development of the 2020 gonorrhea treatment recommendations and the 2021 Centers for Disease Control and Prevention sexually transmitted infection treatment guidelines. Clin Infect Dis 2022;74:S95–111. 10.1093/cid/ciac043 35416971

[6] Unemo M Golparian D Eyre D. Antimicrobial resistance in *Neisseria gonorrhoeae* and treatment of gonorrhea. Methods Mol Biol 2019;1997:37–58. 31119616 10.1007/978-1-4939-9496-0_3

[7] Jacobsson S Cherdtrakulkiat T Golparian D. High susceptibility to the novel antimicrobial zoliflodacin among *Neisseria gonorrhoeae* isolates in eight WHO Enhanced Gonococcal Antimicrobial Surveillance Programme countries in three WHO regions, 2021-2024. IJID Regions 2025;15:100624. 40256400 10.1016/j.ijregi.2025.100624 PMC12008125

[8] Pascual F Au C Chikwari C. Recommendations for the optimal introduction of novel antibiotics to treat uncomplicated gonorrhoea in the face of increasing antimicrobial resistance: a case study with zoliflodacin. BMC Glob Public Health 2024;2:15. 39681965 10.1186/s44263-024-00087-w PMC11622883

[9] Heidary M Ebrahimi Samangani A Kargari A. Mechanism of action, resistance, synergism, and clinical implications of azithromycin. J Clin Lab Anal 2022;36:e24427. 35447019 10.1002/jcla.24427 PMC9169196

[10] Richards DM Heel RC Brogden RN. A review of its antibacterial activity, pharmacological properties and therapeutic use. Drugs 1984;27:469–527. 6329638 10.2165/00003495-198427060-00001

[11] Unemo M Workowski K. Dual antimicrobial therapy for gonorrhoea: what is the role of azithromycin? Lancet Infect Dis 2018;18:486–88. 29523498 10.1016/S1473-3099(18)30162-2 PMC6748319

[12] Adamson P Hieu V Nhung P. Ceftriaxone resistance in *Neisseria gonorrhoeae* associated with the penA-60.001 allele in Hanoi, Viet Nam. Lancet Infect Dis 2024;24:e351–e352. 38723652 10.1016/S1473-3099(24)00230-5 PMC11145020

[13] Balduck M Laumen JGE Abdellati S. Tolerance to ceftriaxone in *Neisseria gonorrhoeae*: rapid induction in WHO P reference strain and detection in clinical isolates. Antibiotics (Basel) 2022;11:1480. 36358135 10.3390/antibiotics11111480 PMC9686967

[14] Programmes, G. H. H. a. S. 2024. Updated recommendations for the treatment of Neisseria gonorrhoeae, Chlamydia trachomatis, and Treponema pallidum (syphilis) and new recommendations on syphilis testing and partner services. Accessed 3 Feburary 2026. https://www.who.int/publications/i/item/9789240090767 39137269

[15] *Clinical treatment of gonorrhea*. 2024. Gonorrhea. Accessed 3 Feburary 2026. https://www.cdc.gov/gonorrhea/hcp/clinical-care/index.html

[16] Taylor SN Marrazzo J Batteiger BE. Single-dose zoliflodacin (ETX0914) for treatment of urogenital gonorrhea. N Engl J Med 2018 Nov 8;379:1835–45. 30403954 10.1056/NEJMoa1706988

[17] Collins JA Basarab GS Chibale K. Interactions between Zoliflodacin and *Neisseria gonorrhoeae* Gyrase and Topoisomerase IV: enzymological Basis for Cellular Targeting. ACS Infect Dis 2024;10:3071–82. 39082980 10.1021/acsinfecdis.4c00438 PMC11320581

[18] Lo F Kong F Hocking J. Treatment efficacy for rectal *Neisseria gonorrhoeae*: a systematic review and meta-analysis of randomized controlled trials. J Antimicrob Chemother 2021;76:3111–24. 34458921 10.1093/jac/dkab315

[19] Perry C Scangarella-Oman N Millns H. Efficacy and safety of gepotidacin as treatment of uncomplicated urogenital gonorrhea (EAGLE-1): design of a randomized, comparator-controlled, Phase 3 study. Infect Dis Ther 2023;12:2307–20. 37751016 10.1007/s40121-023-00862-6 PMC10581980

[20] Bradford P Miller A O’Donnell J. Zoliflodacin: an oral spiropyrimidinetrione antibiotic for the treatment of *neisseria gonorrhea*e, including multi-drug-resistant isolates. ACS Infect Dis 2020;6:1332–45. 32329999 10.1021/acsinfecdis.0c00021

[21] Bhavnani S Cammarata A Hammel J. P-1251. pharmacometric analyses to support dose selection of zoliflodacin, a first-in-class oral antibiotic being developed for the treatment of uncomplicated gonorrhea. Open Forum Infect Dis 2025;12:ofaf000. P-1251.

[22] Unemo M Ahlstrand J Sánchez-Busó L. High susceptibility to zoliflodacin and conserved target (GyrB) for zoliflodacin among 1209 consecutive clinical *Neisseria gonorrhoeae* isolates from 25 European countries, 2018. J Antimicrob Chemother 2021;76:1221–28. 33564854 10.1093/jac/dkab024

[23] Roh KH Luong ND Liu C. In-vitro activities of zoliflodacin and solithromycin against *Neisseria gonorrhoeae* isolates from Korea. Ann Lab Med 2025;45:626–29. 40289852 10.3343/alm.2024.0522 PMC12535836

[24] Luckey A Balasegaram M Barbee LA. 3rd; Zoliflodacin Phase 3 Study Group. Zoliflodacin versus ceftriaxone plus azithromycin for treatment of uncomplicated urogenital gonorrhoea: an international, randomised, controlled, open-label, Phase 3, non-inferiority clinical trial. Lancet 2026;407:147–60. 41391465 10.1016/S0140-6736(25)01953-1 PMC12784215

[25] Basarab GS Kern GH McNulty J. Responding to the challenge of untreatable gonorrhea: ETX0914, a first-in-class agent with a distinct mechanism-of-action against bacterial Type II topoisomerases. Sci Rep 2015;5:11827. 26168713 10.1038/srep11827 PMC4501059

[26] Jacobsson S Golparian D Oxelbark J. Pharmacodynamic evaluation of zoliflodacin treatment of *Neisseria gonorrhoeae* strains with amino acid substitutions in the zoliflodacin target gyrb using a dynamic hollow fiber infection model. Front Pharmacol 2022;13:915346. 10.3389/fphar.2022.874176 PMC9046595 35496288

[27] Lewis D. New treatment options for *Neisseria gonorrhoeae* in the era of emerging antimicrobial resistance. Sexual Health 2019;16:449. 31292063 10.1071/SH19034

[28] Ram S Gill D Rice PA. Combatting antimicrobial-resistant *Neisseria gonorrhoeae*: new antibiotics and the pipeline of antigonococcal therapeutics. Curr Opin Infect Dis 2026;39:36–50. 41452091 10.1097/QCO.0000000000001170

